# Severe hematuria in a patient receiving bevacizumab and pembrolizumab for metastatic cervical cancer: a case report

**DOI:** 10.1186/s12882-023-03101-9

**Published:** 2023-03-10

**Authors:** Yanxin Liu, Changjiang Dong, Xucheng He, Pan Wu, Yamin Shu, Li Chen

**Affiliations:** 1 Department of Pharmacy, West China Second University Hospital, Sichuan University, 610041 Chengdu, Sichuan China; 2 Evidence-based Pharmacy Center, West China Second University Hospital, Sichuan University, Chengdu, China; 3 Key Laboratory of Birth Defects and Related Diseases of Women and Children, Ministry of Education, Chengdu, China; 4 Department of Pharmacy, Pengzhou People’s Hospital, Pengzhou, China; 5 Department of Pharmacy, Pengzhou Second People’s Hospital, Pengzhou, China; 6 Department of Pharmacy, Qionglai Maternal and Child Health and Family Planning Service Center, Qionglai, China; 7 Department of Pharmacy, Tongji Hosiptal, Tongji Medical College, Huazhong University of Science and Technology, Wuhan, China

**Keywords:** Case report, Bevacizumab, Pembrolizumab, Hematuria, Metastatic cervical cancer

## Abstract

**Background:**

Bevacizumab is a monoclonal antibody drug targeting Vascular Endothelial Growth Factor (VEGF), which binds to VEGF receptors to inhibit vascular endothelial cell proliferation and angiogenesis, thus inhibiting tumorigenesis. Pembrolizumab is a monoclonal antibody that can bind to the programmed death-1 (PD-1) receptor, which can block the binding of the PD-1 receptor to its ligands PD-L1 and PD-L2, and release PD-1 pathway-mediated suppression of immune responses. By blocking the activity of PD-1, the purpose of inhibiting tumor growth is achieved.

**Case presentation:**

We report a severe hematuria of bevacizumab plus pembrolizumab, in a 58-year-old woman with metastatic cervical cancer. After three cycles every three weeks of consolidation chemotherapy (carboplatin, paclitaxel, bevacizumab) and following three cycles consolidation chemotherapy (carboplatin, paclitaxel, bevacizumab, pembrolizumab), the patient presented a worsening state. Manifested as massive gross hematuria with blood clots. After stopping chemotherapy, cefoxitin, tranexamic acid and hemocoagulase atrox therapy was administered resulting in rapid clinical improvement. The patient was a cervical cancer with bladder metastasis that increases the risk of development of hematuria. Inhibition of VEGF, which has anti-apoptotic, anti-inflammatory, and pro-survival influences on endothelial cells, weakens their regenerative capacity and increases expression of proinflammatory genes leading to weakened supporting layers of blood vessels and, hence, to damaged vascular integrity. In our patient, the development of hematuria may result from the anti-VEGF effect of bevacizumab. In addition, pembrolizumab may also cause bleeding, and the mechanism of bleeding caused by pembrolizumab is currently unclear, which may be related to immune mediation.

**Conclusion:**

To our knowledge, this is the first case reporting on the development of severe hematuria during bevacizumab plus pembrolizumab treatment, which should alert the clinicians in case of bleeding adverse events onset in older patients under bevacizumab plus pembrolizumab therapy.

## Background

Cervical cancer is the fourth most common cancer type in women and GLOBOCAN appraises of incidence and mortality worldwide for 36 cancers in 185 countries have discovered 569,874 new cases of cervical cancer and 311,365 deaths due to this tumor in 2018 [1]. Primary treatment options include surgery, (chemo)radiation therapy ((C)RT) or systemic chemotherapy. While great advances in preventing cervical cancer by prophylactic vaccinations have been achieved, systemic treatment picking, especially for advanced, metastatic or recurrent cervical cancer, are still restricted [2].

Bevacizumab is a recombinant humanized monoclonal antibody selectively binding to and neutralizing the biological activity of human VEGF.

On 14 August 2014 the Food and Drug Administration (FDA) approved use of bevacizumab in metastatic cervical cancer [3]. It was considered that bevacizumab inhibits cancer growth speculatively impacting only tumor vessels without damaging other vessels [4]. However, in the safety profile of bevacizumab, bleeding is reported among the common adverse events [5].

Pembrolizumab, a monoclonal antibody that blocks the PD-1 receptor presented on T cells, has shown promise as a second-line therapy for PD-L1-positive cervical tumors. Some findings led to the FDA approval of pembrolizumab for the treatment of relapsed or metastatic cervical cancer after frontline chemotherapy treatment, but only for patients whose tumors express PD-L1 or CPS ≥ 1% [6, 7]. Although the emergence of targeted drugs and immune checkpoint inhibition has brought a new dawn of treatment to cancer patients, the accompanying adverse reactions of the whole body and multi-system organs also deserve our attention. There are no cases reported in literature on the adverse reactions caused by combination therapy with bevacizumab and pembrolizumab in patients with metastatic cervical cancer.

To our knowledge, this is the first case reporting on the development of severe hematuria during bevacizumab and pembrolizumab therapy in a patient affected by metastatic cervical cancer. This could be considered as a new risk factor of bevacizumab, which should alert the clinicians when concomitantly receiving pembrolizumab therapy onset in older patients under anti-angiogenic therapy.

## Case presentation

In November 2020, a 58-year-old Chinese female patient with stress incontinence was diagnosed with a cervical adenocarcinoma stage IVA in a Chinese hospital. Her previous family history was negative. But her diagnosis process is tortuous. The patient experienced stress urinary incontinence without obvious incentives in March 2020, no hematuria and other discomforts. She went to a Chinese hospital for urine analysis and B-ultrasound of the urinary system showed no abnormality, and was instructed to observe. In April and July 2020, she went to the doctor twice due to worsening symptoms of urinary incontinence, and there was no other discomfort such as hematuria. B-ultrasound of the urinary system and rectum showed no obvious abnormality in the kidney, bladder, and ureter. She was instructed to continue to observe. In November 2020, she had right lower back pain without obvious incentive, no hematuria, and color doppler ultrasound showed weak echo in the bladder triangle. Considering bladder tumor and urethrovaginal fistula, thereby she underwent bladder tumor diagnostic plasma resection and double lateral ureteral stenting. Until now, the patient’s ureteral stent has not been removed. Postoperative pathological examination revealed adenocarcinoma, and metastasis was not excluded. After consultation with a gynecological oncologist, the physical examination and PET-CT showed an irregular soft tissue mass in the cervical area, which was in line with the diagnosis of cervical cancer. The lesion involved the posterior wall of the bladder, causing it to thicken.

In December 17, she underwent intravenous chemoembolization with cisplatin 90 mg combined with paclitaxel 210 mg of the cervical neoformation after the diagnosis. Later, from January 7, 2021 to February 6, 2021, due to she had tinnitus for seven years, the tinnitus aggravated after chemotherapy, and switched to the carboplatin 500 mg combined with paclitaxel 210 mg for two cycles of chemotherapy, during which the condition was stable. From March 1, 2021 to April 26, 2021, concurrent chemoradiotherapy was performed. But during the period, concurrent chemotherapy was only performed three cycles (the patient had stents in both ureters, little reddish urine after exercise, which could be relieved by rest, and repeated urinary tract infections). She completed a total of twenty-eight external irradiation and six afterloading. In May 17, 2021, MRI scan showed cervical mass, right obturator lymph node and left obturator lymph node were smaller than before, uterine cavity dilatation and vaginal wall thickening were less than before. All of these confirms that her condition has been partially relieved.

In June 5, the patient started consolidation chemotherapy with bevacizumab 400 mg, carboplatin 450 mg, and paclitaxel 210 mg (three cycles every 3 weeks) and started adding PD-1 inhibitor pembrolizumab 200 mg at the fourth consolidation chemotherapy with both good tolerance profile and clinical outcome. In October 8, two days after the 6th cycle of consolidation chemotherapy, the patient suddenly began to experience massive gross hematuria with visible blood clots.

The patient immediately went to a nearest hospital in China and was given cefoxitin 2 g q8h for anti-infective treatment, and tranexamic acid 1 g qd and hemocoagulase 1unit q12h for hemostasis and symptomatic treatment. All of the above treatments are administered by intravenous infusion and were administered for a total of 23 days. The patient was hemodynamically stable on admission and her coagulation indexes were normal before and after hematuria. Unsurprisingly, the patient’s hemoglobin was below 60 g/L, so 1unit of red blood cells was transfused daily for the first three days of starting treatment. The patient’s hemoglobin recovered to above 90 g/L after blood transfusion therapy. The patient’s bleeding was controlled after 10 days of treatment. Then the patient was transferred to a higher-level hospital for further treatment. Her symptoms resolved after 13 days of continued treatment basically. The clinician considered that the previous treatment was effective, so continue to use the original treatment plan. The CT scan showed mass soft tissue shadows were seen in bilateral adnexal areas, the size was about 3.9*2.1 cm, and the bladder wall was unevenly thickened, with a thickness of 2.6 cm. The patient was instructed to suspend chemotherapy, take a 2-week rest for follow-up visits, and permanently discontinue bevacizumab. But in January 2022, the patient suddenly had a small amount of bleeding from the urethral opening after defecation, and slight urinary incontinence. The doctor instructed the patient to temporarily observe, and if the symptoms worsen or the disease progresses, go to the urologist in time. Interestingly, at the same time, the PT/CT scan showed no definite signs of tumor metastasis or recurrence in the body; increased distribution of ^18^ F-FDG in the posterior wall of the bladder, which does not rule out the possibility of blood clots or others; impaired right renal function. During this period, the patient’s appetite decreased significantly, and the rest had no special discomfort. The timeline of the clinical course and interventions of the patient is summarized in Table 1.

**Table 1:** Timeline of clinical course and interventions

Date	Clinical course and interventions
March 2020	**Patient symptoms**: stress incontinence. No relief after observation.
November 2020	**Patient symptoms**: right lower back pain. **Diagnosis**: cervical adenocarcinoma stage IVA.
December 17, 2020- February 6, 2021	**Chemoembolization** with cisplatin 90 mg/carboplatin 500 mg and paclitaxel 210 mg (the paient had tinnitus for seven years, so switch to chemotherapy).
March 4- April 26, 2021	**Concurrent chemotherapy** three cycles with carboplatin 210 mg. **Radiotherapy**: twenty-eight external irradiation and six afterloading.
May 2021	**MRI**: cervical mass, right obturator lymph node and left obturator lymph node were smaller than before, uterine cavity dilatation and vaginal wall thickening were less than before. **Partial response** of the patient’s condition.
June 4- July 29, 2021	**Consolidation chemotherapy** three cycles with carboplatin 450 mg, paclitaxel 210 mg and bevacizumab 400 mg.
August 20- October 6, 2021	**Consolidation chemotherapy** three cycles with carboplatin 450 mg, paclitaxel 210 mg, bevacizumab 400 mg and pembrolizumab 200 mg.
October 8, 2021	**Patient symptoms**: massive gross hematuria with visible blood clots. **Chemotherapy stopped.**
November 8- December 1, 2021	**Symptomatic treatment**: cefoxitin 2 g q8h ivgtt for anti-infective treatment, and tranexamic acid 1 g qd ivgtt and hemocoagulase 1unit q12h ivgtt for hemostasis and symptomatic treatment (23 days). **Clinical improvement in 10 days.**
November 2021	**CT**: mass soft tissue shadows were seen in bilateral adnexal areas, the size was about 3.9*2.1 cm, and the bladder wall was unevenly thickened, with a thickness of 2.6 cm.
January 2022	**Patient symptoms**: a small amount of bleeding from the urethral opening after defecation, and slight urinary incontinence.
January 2022	**PT/CT imaging**: no definite signs of tumor metastasis or recurrence in the body; increased distribution of 18 F-FDG in the posterior wall of the bladder, which does not rule out the possibility of blood clots or others; impaired right renal function. **Stable disease** of the patient’s condition.

In addition, thrombocytopenia may cause bleeding in the gums, nasal cavity, skin and mucous membranes, and in severe cases, bleeding from internal organs, head and urinary system may occur, which is life-threatening [8]. Fortunately, the clinical pharmacist monitored the patient’s platelet status, and she has platelet abnormalities only after massive blood loss and this may be related to the patient’s heavy bleeding. Therefore, patients are actively given platelet-raising drug interleukin-11 support therapy and back to normal quickly (Table 2).

**Table 2:** Results of platelet count

Date	Chemotherapy Course	PLT (× 10^12^/L)
December 17, 2020	first chemotherapy	282
January 7, 2021	second chemotherapy	264
February 6, 2021	third chemotherapy	252
April 4, 2021	first concurrent chemoradiotherapy	138
April 11, 2021	second concurrent chemoradiotherapy	220
April 17, 2021	third concurrent chemoradiotherapy	184
June 4, 2021	first consolidation chemotherapy	202
June 29, 2021	second consolidation chemotherapy	241
July 29, 2021	third consolidation chemotherapy	203
August 20, 2021	fourth consolidation chemotherapy	201
September 13, 2021	fifth consolidation chemotherapy	178
October 6, 2021	sixth consolidation chemotherapy	208
October 31, 2021	NA	53
November 11, 2021	NA	191

## Discussion and conclusions

This case report suggested that elderly patients with cervical cancer with bladder metastases and a history of post-active hematuria may be a high-risk factor for bevacizumab plus pembrolizumab use and deserve more attention from doctors, nurses, and pharmacists. There are two main types of bleeding caused by bevacizumab. One is skin and mucous membrane bleeding, which is usually grade 1 bleeding from the gums, vagina, and nasal mucosa. Gastrointestinal bleeding (melena, rectal bleeding) in cancer patients and emoptysis and pulmonary hemorrhage in non-small cell lung cancer patients [9]. In addition, bevacizumab-induced hemorrhage and urinary tract hemorrhage in patients with urological tumors are rare but relatively critical. The total proportion of grade 3 or higher bleeding events is 0.4-5%, and bevacizumab treatment should be permanently discontinued if grade 3 or 4 bleeding occurs [10]. The decision to resume treatment with bevacizumab should be taken with great care and based on the patient’s clinical stability, and multi-disciplinary discussion of the risk–benefit ratio. After the patient developed gross hematuria and urinary tract infection, the clinician gave the patient symptomatic and supportive treatment as soon as possible, and instructed to suspend chemotherapy and permanently discontinue bevacizumab.

In our patients, the development of hematuria may attribute to the anti-VEGF effect of bevacizumab. As a neutralizing antibody against VEGF, bevacizumab may undermine vascular integrity by the inhibition of endothelial survival and proliferation. More importantly, bevacizumab may indirectly lead substantial undermine to the vascular wall infiltrated with cancer cells by having an enhanced cytotoxic effect on tumors. This could be one of the reasons for the hematuria in our patient [11]. Finally, bevacizumab may lead vascular undermine through the so-called ‘radiation recall phenomenon’ in patients with previous radiation therapy, as suggested by a case report [12]. Our patient had also received radiation therapy, which may have been another cause of hematuria.

Moreover, thrombocytopenia may cause bleeding in the gums, nasal cavity, skin and mucous membranes, and in severe cases, bleeding from internal organs, head and urinary system may occur, which is life-threatening [8]. Platelets are the carrier of VEGF, and inhibition of VEGF by bevacizumab can directly lead to platelet dysfunction and interfere with hemostasis [13]. Thus, for patients using bevacizumab, clinicians and pharmacists should also attach great importance to the thrombocytopenia of patients, and be cautious about the occurrence of thrombocytopenia to prevent more serious consequences. In our patient, thrombocytopenia only occurs after the patient has lost a lot of blood, thus we can rule out that her hematuria is due to thrombocytopenia.

But, why do we attribute the patient’s hematuria to the adverse event of the combination of bevacizumab and pembrolizumab? First, the occurrence of hematuria was time-correlated with the use of bevacizumab and pembrolizumab. Our patient developed hematuria 2 days after three courses of bevacizumab alone and three courses of both agents. Second, when hematuria occurs, the patient’s condition is in a stable phase and undergoing regular chemotherapy. It is unlikely the hematuria due to an underlying disease but the possibility of tumor-related bleeding cannot be ruled out. Finally, other possible causes of hematuria, such as coagulation abnormalities, thrombocytopenia, and cardiovascular disease, have been ruled out by doctors. In conclusion, we believe that the combined use of bevacizumab and pembrolizumab may cause hematuria in our patient. However, the use of pembrolizumab may also induce or exacerbate hematuria.

It has been reported that pembrolizumab may also cause bleeding, including epistaxis, oral bleeding, gastric bleeding, and hematuria, but the incidence is low [14–16]. In addition, studies have shown that combination therapy will show a higher incidence of adverse events than single drugs, which may be due to different principles of adverse events caused by different drugs. For example, pembrolizumab activates the immune function of immune cells, making immune cells have an enhanced attack effect on tumor cells [17], and at the same time cause damage to healthy cells and tissues. When chemotherapy drugs or targeted drugs are used in combination, corresponding cytotoxicity is introduced. The adverse reactions caused by the two principles are superimposed, which increases the probability and severity of adverse reactions. Although the instructions of pembrolizumab clearly pointed out that it has adverse reactions of bleeding, the mechanism is currently unclear. There are few reports in the literature that pembrolizumab may cause adverse effects related to bleeding disorders [14–16, 18]. However, the study showed that hematuria was associated with a significantly higher risk of chronic kidney disease (CKD) progression and death in a large adult cohort with CKD [19]. This appears to be validated in our patients as well. January 2022 PET-CT scan showed that impaired right kidney function. The previous medical examinations had never suggested abnormal kidney function in the patient. Therefore, clinicians should be extra vigilant in the patients who was treatment with bevacizumab plus pembrolizumab.

At present, there are few clinical trials exploring the safety of bevacizumab in combination with pembrolizumab. Research has shown that the combination of 200 mg of pembrolizumab and a 15 mg/kg dose of bevacizumab given every 3 weeks is safe and active in metastatic renal cell carcinoma [20]. Another phase 2 nonrandomized clinical trial showed that the combination of pembrolizumab with bevacizumab was well tolerated [21]. The most common adverse event reported in previous studies was hypertension. Until now, there is no clinical trial report on the combined application of the two drugs causing bleeding. This may be due to the limited follow-up time in clinical trials. Also, it is rare for a combination of two drugs to cause bleeding. Thus, to our knowledge, this is the first case reporting on the development of severe hematuria during bevacizumab and pembrolizumab therapy in a patient affected by metastatic cervical cancer.

Our patient had tolerated bevacizumab well prior to undergoing pembrolizumab therapy and developed her hematuria only after pembrolizumab three cycles (which she initially tolerated well). As targeted therapy and immunotherapy becomes more accepted in the treatment of cervical cancer, our case emphasizes the importance of monitoring for symptoms that could be attributed to adverse events from immunotherapy after targeted therapy. We speculate that the combination of bevacizumab and pembrolizumab may be one of the high-risk factor for hematuria in this patient. However, more clinical trials and case reports are still needed to confirm this speculation.

This new bevacizumab and pembrolizumab related adverse event should alert the clinicians in case bleeding symptoms develop in older patients under chemotherapy and should lead to further investigations. In regard to our patient, prompt hemostasis and anti-infective therapy resolved acute symptoms, and no urinary symptom worsening was observed during the three months of follow-up. She will continue oncological and urinary follow-ups. Besides, she siad, the long treatment process of the tumor has caused huge damage to my body, mind and economy. Now, hematuria has made my life very inconvenient. From the initial shock to the long-term hospitalization, although my condition has entered a stable stage, I still spend every day in anxiety and depression.

To sum up, patients should be carefully inquired about their medical history before clinical application of bevacizumab, and it should be used with caution in patients with bladder metastases or combined with urinary tract tumors, especially if patients have active hematuria before use. More caution should be exercised when using pembrolizumab in combination, and patients should be closely monitored for bleeding such as subcutaneous ecchymosis and petechiae. In addition, many tumor patients have bone marrow suppression problems such as thrombocytopenia. When using bevacizumab, close attention should be paid to the patient’s platelet status.

## Data Availability

Records and data pertaining to this case are in the patient’s secure medical records in the West China Second University Hospital, Sichuan University. If needed, the relevant material can be provided by corresponding author on reasonable request.

## Abbreviations

VEGF: Vascular endothelial growth factor
PD-1: Programmed death-1
(C)RT: (Chemo)radiation therapy
FDA: Food and drug administration
CKD: chronic kidney disease
